# Xanthogranulomatous Breast Mass: An Unusual Presentation

**DOI:** 10.7759/cureus.17973

**Published:** 2021-09-14

**Authors:** Ibtesam G Zahid, Suryanaren Kummarapurugu, Sameer Alrefai

**Affiliations:** 1 General Surgery, Edward Via College of Osteopathic Medicine, Blacksburg, USA; 2 General Surgery, Sentara Halifax Regional Hospital, South Boston, USA

**Keywords:** breast cancer pathology, xanthogranulomatous epithelial tumor, touton giant cells, general surgery and breast cancer, rare breast mass, xanthogranulomatous reaction

## Abstract

Xanthogranulomatous inflammatory reactions are benign inflammatory processes characterized by aggregating lipid-laden foamy macrophages. Although cases have been reported in different organ systems, these rare reactions predominantly occur in the kidney and gallbladder. We present a 92-year-old female who noticed a palpable, tender mass in the lower inner quadrant of her right breast with no skin changes. She was referred to surgery by her primary care physician on suspicion of malignancy and further evaluation. Ultrasound-guided biopsy, ordered by the primary care provider, revealed a suspicious high-grade malignant neoplasm of uncertain origin. Pathological findings include the presence of an unusual population of malignant epithelioid cells with a striking xanthogranulomatous reaction, along with numerous Touton-like histiocytes. These findings are comparable in morphology to a recently reported xanthogranulomatous epithelial tumor. Given the lack of history of breast carcinoma in this patient as well as the lack of immunohistochemical studies suggesting breast carcinoma, treatment involved continuing standard of care for an unusual high-grade sarcoma via lumpectomy. A positron emission tomography (PET) scan was ordered to ensure there was no spread or alternate origins of the cancer tissue. This case report brings to light the findings of a probable xanthogranulomatous tumor in breast tissue, an exceptionally rare phenomenon in breast cancer, especially in the elderly population. Due to the rarity of xanthogranulomatous tumors in the breast, prognosis and standardized treatment have yet to be established.

## Introduction

Breast cancer is one of the most common cancers among women in the United States (US) [1]. The most common categories of breast cancer are infiltrating ductal carcinoma and lobular carcinoma. In this case, an unusual breast tumor was discovered in an elderly patient, with distinctive histologic features that have previously not been documented. These include weakly keratin-positive epithelial cells with malignant cytologic features. This tumor also had characteristic features of a xanthogranulomatous reaction [2] and was immunohistochemically negative for several key markers mentioned in the discussion.

Xanthogranulomatous inflammatory reactions are benign inflammatory processes characterized by aggregating lipid-laden foamy macrophages. These reactions predominantly occur in the kidney and gallbladder [3,4] and are generally triggered by abscesses, necrosis, and hemorrhage. They are characterized by eosinophilic, granular, periodic acid-Schiff (PAS)-positive histiocytes in initial stages, followed by foamy macrophages, activated plasma cells, suppurative foci, and hemorrhages [2]. Eventually, these lesions and neoplasms become fibrotic. Recently, epithelial tumors have been identified in soft tissue and bone in six cases that presented with features of xanthogranulomatous inflammation. These cases involved young patients, and the tumors behaved like low-grade neoplasms [5]. 

We present a case report of a patient with a right breast mass that was inconsistent with previously documented histological and pathological features of breast cancer. Rather, it had characteristics consistent with xanthogranulomatous epithelial tumors. Because of the tumor’s close resemblance to carcinoma with tumor cells, comprised of abundant cytoplasm and prominent nucleoli, it introduced challenges with regards to the treatment approach. Due to lack of tumor extension into surrounding tissues, and negative immunohistochemical studies, treatment was continued under the notion that this tumor was an unusual high-grade sarcoma [6].

## Case presentation

An elderly 92-year-old female noticed a palpable mass in the lower inner quadrant of her right breast for several months. The patient reported occasional palpable tenderness but no skin changes, nipple retraction, or discharge. She denied any family history of breast cancer. 

**Figure 1:**
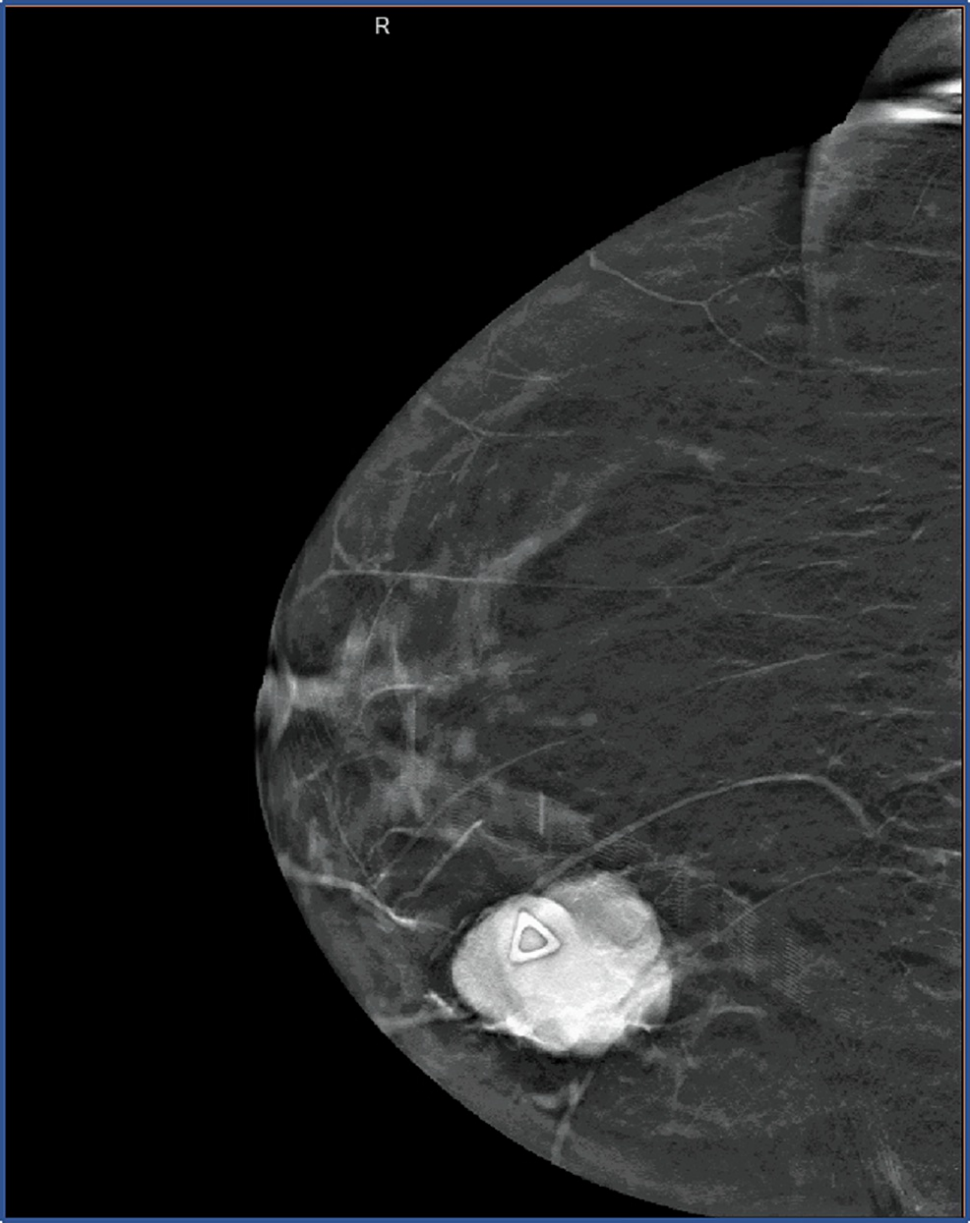
Mass visualized on mammogram before excision. Tumor measured 5.0 x 3.5 x 3.0 cm upon excision

A mammogram was performed and demonstrated a suspicious right breast mass (Figure 1). It was followed up with ultrasound-guided core-needle breast biopsy, which further showed a large, lobulated mass corresponding to the mammographic findings suspicious for a malignant neoplasm, Breast Imaging-Reporting and Data System (BI-RADS) category 4 [7].

Surgery was consulted, subsequent right breast lumpectomy and sentinel lymph node biopsy were performed. Pathology reports demonstrated malignant tumor cells with unknown etiology most likely consistent with a soft tissue mass and no lymph node involvement. She underwent a positron emission tomography (PET) scan that showed no distant metastases or axillary uptake. The patient declined any further treatment and continued to follow up with oncology with consideration for bilateral mammograms in the future. 

## Discussion

This inflammatory process of a xanthogranulomatous reaction is a rare pathological finding; however, it has been described in many organ systems including the gallbladder, kidneys, and ovaries. This reaction causes a diffusely destructive process that can be compared to chronic cholecystitis in the gallbladder but mimicking gallbladder carcinoma [8]. Xanthogranulomatous pyelonephritis, also diagnosed by histology, has also shown similar features to that of renal cell carcinoma [9]. Breast involvement is limited to a few cases of mastitis and can be described as a solid lump masquerading as a malignancy [10]. 

**Figure 2:**
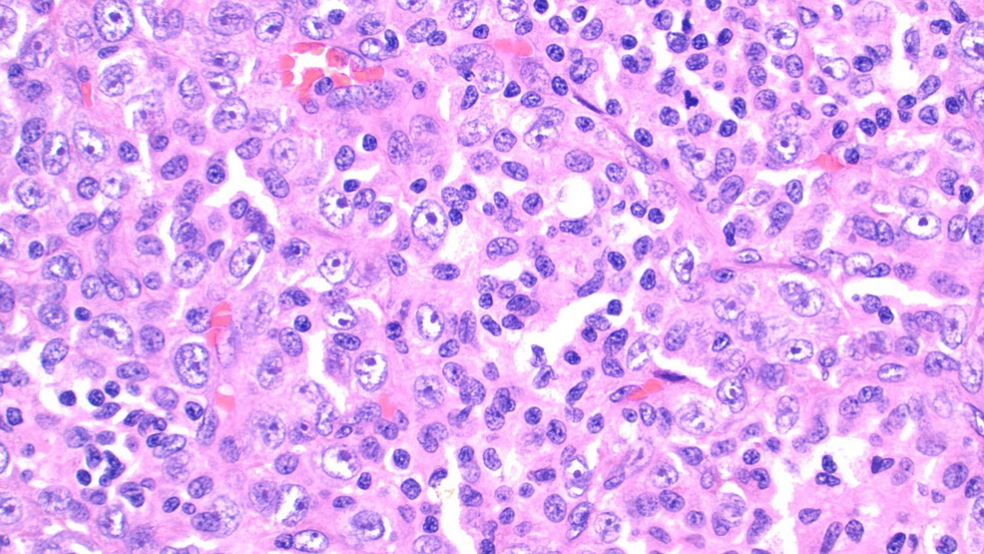
Malignant-appearing epithelioid cells with abundant cytoplasm and prominent nucleoli with nests of foamy histiocytes mixed with lymphocytes and scattered neutrophils.

**Figure 3:**
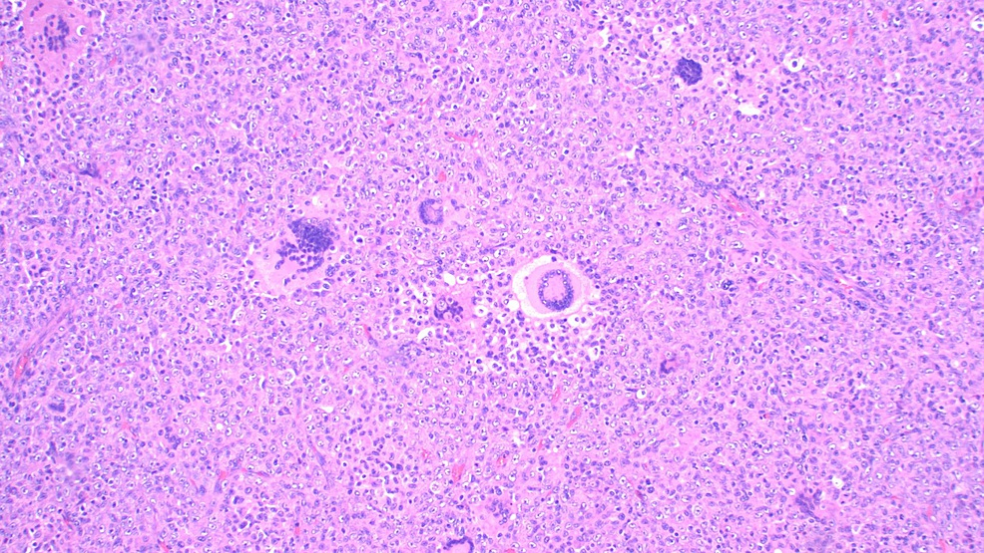
Giant Touton-like histiocytes comprised of multinucleated cells.

**Figure 4:**
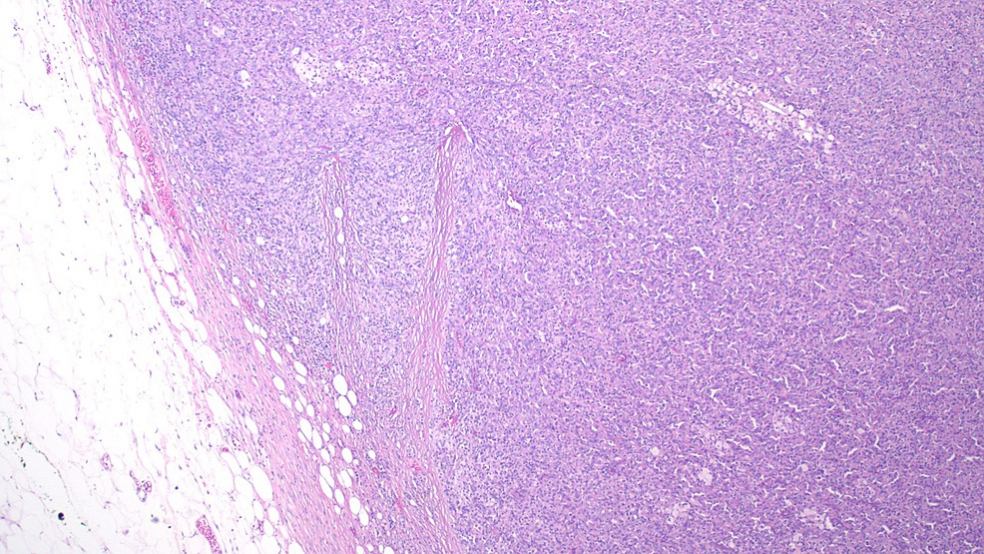
Non-malignant fatty breast tissue on the left adjacent to the fibrous capsule of xanthogranulomatous tumor containing lymphoid aggregates with lipid-laden foamy histiocytes.

**Figure 5:**
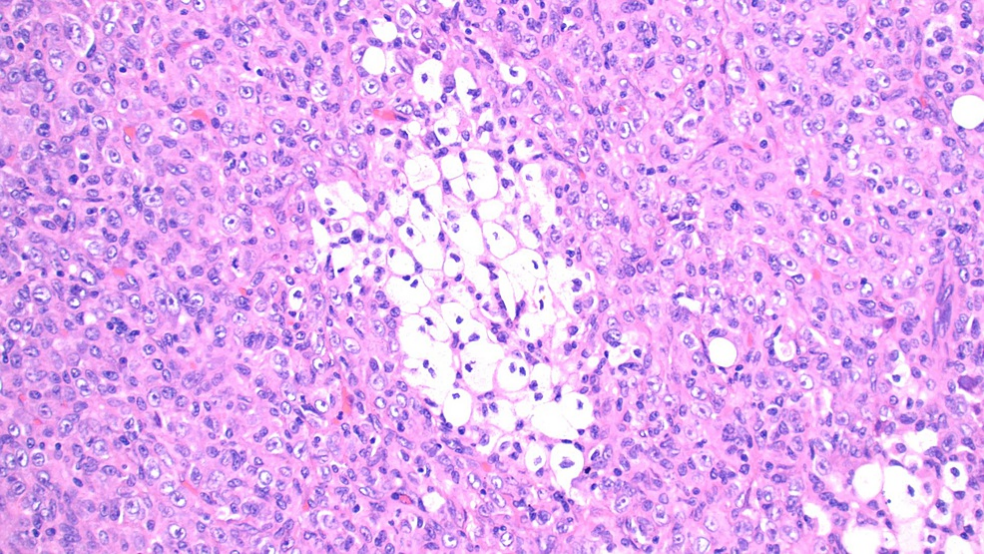
Lipid-laden necrotic reaction surrounded by foamy histiocytes.

Pathological analysis of the tumor, in this case, displayed an unusual population of malignant-appearing epithelioid cells with a striking xanthogranulomatous reaction including aggregating lipid-laden foamy histiocytes (Figure 2) and numerous Touton-like histiocytes (Figure 3) surrounded by a fibrous capsule (Figure 4). Another striking feature of xanthogranulomatous inflammation included a lipid-laden necrotic reaction (Figure 5). These features, while rare, were comparable in morphology to a recently reported xanthogranulomatous epithelial tumor. Additionally, the tumors seemed to behave as low-grade neoplasms in a younger population (16-62 years) [5].

By immunohistochemistry, many of the malignant-appearing epithelioid cells are weakly positive for keratins with the AE1/AE3 antibody and epithelial membrane antigen. Signal transducer and activator of transcription 6 (*STAT6*), transducin-like enhancer of split 1 (*TLE1)*, high molecular weight keratin, CAM 5.2, S100 protein, OSCAR keratin, CD3, CD20, CD30, CD45, CD68, smooth muscle actin, and desmin are negative in these cells. Very focal reactivity was shown to estrogen receptors (ER) with negative results for progesterone receptors (PR), pankeratin, melanoma antigen recognized by T cells (Mart-1), and Human Melanoma Black (HMB-45) [11]. CD163 is positive in background histiocytes. Integrase interactor 1 (*INI1*) and Brahma-related gene-1 (*BRG1*) expression are retained. Keratin 7 is negative. While this tumor bears striking resemblance to the xanthogranulomatous epithelial tumor, it is difficult to rule out the possibility of an exceptionally unusual carcinoma. The unusual histological findings, especially in the breast, present a challenge in determining the true etiology of this inflammatory process and whether it is a true xanthogranulomatous epithelial tumor or a unique incidental inflammatory finding. Given the lack of history of breast carcinoma in this patient as well as the lack of immunohistochemical studies suggesting breast carcinoma, treatment involved continuing standard care for an unusual high-grade sarcoma via lumpectomy [6]. Wide clear margins were obtained and the patient refused further care probably due to her advanced age and unwillingness to undergo further treatment such as chemotherapy or radiation to prevent recurrence but continued to follow with oncology.

## Conclusions

This case report brings to light the findings of a probable xanthogranulomatous tumor in breast tissue; an exceptionally rare phenomenon in breast tissue, especially in the elderly population. Although most xanthogranulomatous reactions represent benign processes, more aggressive forms can present as seen in this case. Due to the rarity of xanthogranulomatous tumors in the breast, prognosis and a standardized treatment including further surveillance and follow-up have yet to be established. 

## References

[1] Coughlin SS (2019). Epidemiology of breast cancer in women. Adv Exp Med Biol.

[2] Cozzutto C, Carbone A (1988). The xanthogranulomatous process. Xanthogranulomatous inflammation. Pathol Res Pract.

[3] Siddappa S, Ramprasad K, Muddegowda MK (2011). Xanthogranulomatous pyelonephritis: a retrospective review of 16 cases. Korean J Urol.

[4] Kansakar PB, Rodrigues G, Khan SA (2008). Xanthogranulomatous cholecystitis: a clinicopathological study from a tertiary care health institution. Kathmandu Univ Med J (KUMJ).

[5] Fritchie KJ, Torres-Mora J, Inwards C (2020). Xanthogranulomatous epithelial tumor: report of 6 cases of a novel, potentially deceptive lesion with a predilection for young women. Mod Pathol.

[6] Hsu C, McCloskey SA, Peddi PF (2016). Management of breast sarcoma. Surg Clin North Am.

[7] Balleyguier C, Ayadi S, Van Nguyen K, Vanel D, Dromain C, Sigal R (2007). BIRADS classification in mammography. Eur J Radiol.

[8] Ewelukwa O, Ali O, Akram S (2014). Xanthogranulomatous cholecystitis mimicking gallbladder cancer. BMJ Case Rep.

[9] Moss BF, Potter L, Cliff A, Kumar M (2019). Xanthogranulomatous pyelonephritis with associated renal cell carcinoma. BMJ Case Rep.

[10] Hussain T, Elahi B, Long E, Mahapatra T, McManus PL, Kneeshaw PJ (2012). Xanthogranulomatous inflammation involving latissimus dorsi donor site and implant breast reconstruction: case report and literature review. World J Surg Oncol.

[11] (2021). Stains (IHC & special) & CD markers. https://www.pathologyoutlines.com/stains.html.

